# Cardiorespiratory and metabolic responses to body mass-based squat exercise in young men

**DOI:** 10.1186/s40101-017-0127-9

**Published:** 2017-02-08

**Authors:** Miki Haramura, Yohei Takai, Takaya Yoshimoto, Masayoshi Yamamoto, Hiroaki Kanehisa

**Affiliations:** 1 Sports and Life Science, National Institute of Fitness and Sports in Kanoya, 1 Shiromizu, Kanoya, Kagoshima 891-2393 Japan; 2 Japan Institute of Sports Sciences, 3-15-1 Nishigaoka, Kita-ku Tokyo, 115-0056 Japan

**Keywords:** Resistance exercise, Lactate threshold, Oxygen uptake, Aerobic metabolism, Electromyograms

## Abstract

**Background:**

The purpose of this study was to quantify cardiorespiratory and metabolic responses to body mass-based squat exercise, with specific emphasis on the relationships with the exercise duration.

**Methods:**

Fifteen healthy young men performed body mass-based squat exercise as well as an incremental loaded bicycle test, which determine maximal oxygen uptake and maximal heart rate, with an interval of 2 days between the tests. During both tasks, oxygen uptake, blood lactate concentration (BLa), and heart rate (HR) were determined. Oxygen uptake in both tasks was divided by body mass (VO_2_). VO_2_ in the squat task was normalized to VO_2_ in the incremental test (%VO_2_max). In addition, electromyograms (EMGs) were also recorded from the vastus lateralis, rectus femoris, vastus medialis, biceps femoris, and gluteus maximus.

**Results:**

Cardiorespiratory parameters and BLa did not change after 5 min from the exercise onset. The %VO_2_max and BLa during body mass-based squat exercise were significantly related to maximal VO_2_ obtained by the incremental test. Metabolic equivalents reached 6.5 when the squat exercise was continuously performed for 5 min.

**Conclusions:**

These findings indicate that (1) the squat exercise adopted here is of moderate intensity and predominantly uses aerobic energy supply after 5 min from the start of the exercise and (2) relative intensity during the exercise depends on an individual’s maximal aerobic power.

## Background

It is well known that a training program consisting of many repetitions with low load is an effective maneuver for developing local muscular endurance [1]. Recently, Miyamoto et al. [2] have observed that 4 weeks of low-intensity electrical muscle stimulation into the knee extensors improved peak oxygen uptake and ventilatory threshold during incremental bicycle exercise. This suggests that low intensity and high repetition resistance training can enhance not only local but also systemic endurance capacities. In addition, many studies have tried to determine lactate threshold (LT) at which glycolytic metabolism is enhanced and energy supply shifts from aerobic to glycolytic metabolism [3], by using incremental resistance exercises of the lower extremities [4–8]. It has been shown that LT intensities during half squats [8] and leg press [4–6] exercises were 25 and 27% of one repetition maximum (1RM), respectively. When the squat exercise at the corresponding intensity is conducted continuously for 30 min (30 s active with an interval of 1 min × 20 sets), respiratory response and blood lactate concentration during the exercise appear to be stable after 3 to 6 min from task onset, indicating that squat exercise at LT intensity is predominantly aerobic in nature [6]. Exercise at LT intensity can be conducted continuously without blood lactate accumulation because of equilibrium between the rate of lactate appearance into and disappearance from the blood occurs, reflecting a predominance of aerobic metabolism [9, 10].

It is known that body mass-based exercise training (e.g., squat, calf raise, and hip flexion exercises) is feasible and effective for improving muscular strength of the knee extensors in elderly populations [11, 12] and percent body fat, muscular strength of the knee extensors, and jump performance in adolescences [13, 14]. A training scheme consisting of body mass-based exercise has some advantages in that everyone can perform such exercises anywhere with no special apparatus. However, prior studies have provided less information concerning cardiorespiratory and metabolic responses to body mass-based squat exercise. Isear et al. [15] have reported that, for young men, activity level of the vastus lateralis during body mass-based parallel squat exercise was 33% of that observed during maximal voluntary contraction. Taking this into account together with the reports of Garnacho-Castano et al. [8] and de Sousa et al. [6], it may be assumed that, at least for young adults, body mass-based squat exercise would be predominately supported via aerobic metabolism when it is performed continuously.

The purpose of this study was to quantify cardiorespiratory and metabolic responses to body mass-based squat exercise, with specific emphasis on the association with the exercise duration. To this end, we mainly focused the exercise times at which the measured variables reached the stable conditions and the magnitude of the measured variables at the corresponding times. As mentioned above, the previous studies that examined the effects of body mass-based exercise training have focused on improvement in muscle function and body composition. The findings obtained here will be useful for discussing whether or not body mass-based exercise can be a training modality for improving not only muscular endurance but also cardiorespiratory function.

## Methods

### Subjects

Fifteen healthy men (age, 24.2 ± 4.8 years; height, 171.2 ± 5.1 cm; body mass, 65.4 ± 7.4 kg; percent body fat, 16.0 ± 4.4%, means ± SDs) participated in this study. Physical characteristics of the subjects are presented in Table 1. All subjects were involved in physical activities such as jogging and cycling for at least 3 h per week, but they did not perform regular resistance training. All subjects were free of long-term use of oral steroids use or other medications that can influence weight gain, multiple food allergies, moderate or substantial physical or developmental disability, or infection. They refrained from eating, smoking, or drinking tea or coffee for 2 h prior to the test. This study was approved by the ethical committee of the National Institute of Fitness and Sports in Kanoya. Prior to the experiment, all subjects were informed of the purpose and procedures of the study and possible risks. Written informed consent was obtained from all subjects.

**Table 1:** Physical characteristics of subjects

Age, years	24.2 ± 4.8
Body mass, kilograms	65.4 ± 7.4
Height, centimeters	171.2 ± 5.1
Percent body fat, %	16.0 ± 4.4
KET/BM, newton-meter per kilogram	3.0 ± 0.9
BLa at LT, millimoles per liter	2.3 ± 0.5
%VO_2_max at LT, %	46.5 ± 6.2
%HRmax at LT, %	65.8 ± 4.8

Data are presented as means ± SDs

*KET/BM* knee extension torque relative to body mass, *BLa* blood lactate concentration, *LT* lactate threshold, *%VO*_*2*_ *max* oxygen uptake during squat exercise relative to maximal oxygen uptake, *%HRmax* heart rate during squat exercise relative to maximal heart rate

### Experimental design

Subjects participated in two experimental sessions with an interval of 2 days between sessions. An incremental loaded bicycle test was conducted to determine maximal oxygen uptake (VO_2_max) and maximal heart rate (HRmax) in the first session. In the second session, subjects continuously performed 200 times body mass-based squat exercises. Respiratory gas, heart rate, blood lactate concentration, knee joint angle, and muscular activity were measured during both sessions.

### Incremental loaded bicycle test

The incremental test was conducted in accordance with the procedure used by James et al. [16], using an electrically braked bicycle ergometer (COMBI, AEROBIKE75XLIII, Tokyo, Japan). After a 5-min rest, subjects pedaled the bicycle with an initial load of 75 W. The load was incrementally increased by 25 W every 3 min until exhaustion. Pedaling frequency was held constant at 60 rpm with the aid of an audible metronome. We set the following criteria to judge termination of the test: (1) oxygen uptake was at a steady state, (2) rating of perceived exhaustion (RPE) was 19 or 20, (3) subjects were unable to maintain a pedaling rate of 60 rpm, (4) respiratory exchange ratio (RQ) >1.15, and (5) heart rate was at steady state (estimated value ± 15 bpm) [17].

### Body mass-based squat exercise

Subjects continuously performed a body mass-based squat exercise, completing 200 repetitions. The squat task was conducted at a tempo of 45 rpm (2 beats/time) which allows subjects to carry it out correctly [18]. Subjects stood with their legs to shoulder width and squatted with the knee joint flexed at 90° from standing position, and then returned to initial position. A box was put behind the subjects to control range of motion during the squat task. Subjects were asked to pull their hip behind, and not to lean their trunks forward and move their knees forward, and stop the motion when changing from ascending (descending) to descending (ascending) phases during the task.

### Respiratory gas

During the tasks, respiratory gas was collected continuously to determine oxygen uptake (VO_2_), carbon dioxide production (VCO_2_), minute ventilation (VE), and respiratory exchange ratio. Before the experiment commenced, flow volume calibration and oxygen gas calibration were performed using an automated breath-by-breath system that was previously calibrated (Vmax Specrta 229, Sensor medics Corp., Yorba Linda, CA, USA). During the incremental test, VO_2_max was taken to be the mean VO_2_ over the final 30 s of the incremental phase [19]. During the squat exercise, respiratory gas data was averaged for each 1 min epoch. VO_2_ during both tasks was normalized to body mass. The VO_2_ during the squat task was normalized to VO_2_max and expressed as relative value (%VO_2_max). Metabolic equivalents (METs) during the body mass-based squat exercise were calculated from metabolic rate at rest (3.5 ml/kg/min) [20, 21] and VO_2_ during the squat exercise.

### Heart rate

Heart rate (HR) was monitored every 1 s using telemetry (Polar RC3 GPS, Polar Electro OY; Kempele, Finland). HR was averaged over every 1 min, and normalized to HRmax, and expressed as relative value (%HRmax).

### Blood lactate concentration

Blood sample (0.3 μl) was collected from the fingertip using a portable digital Lactate Pro meter (Lactate Pro2, LT-1730, ARKRAY Factory Inc, Kyoto, Japan) and in the last 30 s of each stage during the incremental load test. Blood lactate concentration (BLa) was averaged over every 1 min during the squat exercise. The LT was estimated using the algorithm adjustment method [5] and based on the procedure described by Orr et al. [22], as work intensity at which lactate concentrations start to increase in an exponential manner [23].

### Knee joint angle

To determine the ascending and descending squat exercise phases, knee joint angles were recorded using an electronic goniometer (SG-150, Biometrics, Gwent, UK). The goniometer was attached to the lateral side of the thigh and lower leg with adhesive tape. Joint angle signals were recorded and stored via a 16-bit analog/digital converter (PowerLab/16S, ADInstruments, Sydney, Australia) on a personal computer at a sampling frequency of 2 kHz.

### Electromyogram

During both maximal voluntary contraction (MVC) and squat tasks, surface electromyograms (EMGs) (ME6000T, MEGA Electronics, Finland) with Ag-AgCl electrodes (diameter 10 mm; interelectrode distance 20 mm) (N-00-S, Blue Sensor M, Ambu, Denmark) were recorded from the vastus lateralis (VL), rectus femoris (RF), vastus medialis (VM), biceps femoris (BF), and gluteus maximus (GM) muscles from the right leg. After the skin surface was shaved, rubbed with sandpaper, and cleaned with alcohol, the electrodes were attached to the skin over the muscle belly to the direction of fascicles at the same location between days, according to the method reported by Tillin et al. [24]. The electrode locations were at 55% (VL), 50% (RF), 90% (VM), and 45% (BF) of the distance between the greater trochanter and the lateral femoral condyle. The location of each electrode was marked on the skin surface with a permanent maker. A ground electrode (preamplifer) was attached near the two electrodes. EMG signals were collected at a sampling frequency of 2 kHz and stored on a personal computer. EMG signals and MVC torques is obtained from the right side. To normalize muscular activity during the squat task, the subject exerted MVC in each muscle. For the MVC tasks, subjects gradually exerted each torque from baseline to maximum and then sustained at maximum for approximately 2 s. Subjects performed two trials, with a 3-min interval between trials. To determine maximal EMG amplitudes of the knee extensors and flexors, subjects exerted knee extension and flexion torques with a dynamometer (Biodex System 2; Biodex Medical Systems, NY, USA). Subjects were fixed on an adjustable chair with hip and knee joints flexed at 90°. To prevent the joint angle from changing during the tasks, the trunk and hips were fixed using non-elastic belts. To quantify maximal GM EMG amplitude, subjects produced hip extension torque using a custom-made device (AO-2T, Applied Office, Japan) equipped with tension/compression load cells (LUR-A-SA1, Kyowa, Japan). Subjects lay down prone on a bed with their knee joint flexed at 90°. To prevent the joint angle from changing during the task, the trunk and hips were fixed by non-elastic belts. An additional trial was conducted if the difference in the peak values between the trials was more than 10%. The trial with the highest peak force was adopted for further analysis. MVC torques were normalized to body mass.

**Fig. 1:**
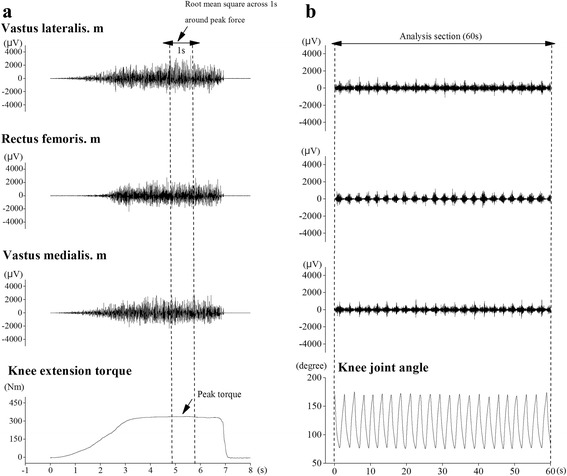
Example data of the MVC task data for knee extension (**a**) and body mass-based squat exercise (**b**)

Root-mean-square (RMS) was calculated from the EMG amplitudes of each muscle during the MVC and squat tasks, using data analysis software (Chart version 7; ADInstruments, Australia). In the MVC task, the RMS value (EMG_MVC_) was determined over a 1-s window centered at the time at which peak torque was attained (Fig. 1a). In the squat task, the RMS value was determined over a 1-min window (Fig. 1b). The RMS value during the squat task was normalized to EMG_MVC_ and expressed as a relative value (%EMG_MVC_).

In our preliminary study, seven young men performed MVCs twice with an interval of 7 days to confirm the reproducibility of MVC measurements. As the results, intra-class correlation coefficients were ≥0.88 for MVC torques, and ≥0.79 for EMG amplitudes during the MVCs, satisfying a criteria for reproducibility of the measurement (>0.75) [25].

### Statistical analysis

Descriptive data are presented as means ± SDs. Prior to the experiment, we have estimated sample size based on Cohen’s criteria (effect size, 0.40; an α level, 0.05; a power (1-β), 0.95) [26] by using statistical software (G*Power 3.1.9.2, Heinrich-Heine-Universitat Düsseldorf, Düsseldorf, Germany; http://www.gpower.hhu.de/). The results revealed that at least nine subjects were necessary as the total sample size. Independent variables were VO_2_, %VO_2_max, METs, VE, RQ, HR, %HRmax, BLa, and %EMG_MVC_. Mauchly’s test of sphericity was used to confirm variable homogeneity. To test for significant time-related differences in these variables during the body mass-based squat exercise, one way analysis of variance (ANOVA) with repeated measures was used. When appropriate, Bonferroni tests were used for post hoc comparisons. In this study, we defined the condition in which the measured variable did not significantly differ between adjacent time points as stable condition. Pearson’s product-moment correlation coefficient (*r*) was calculated for relationships between VO_2_max and each of BLa and %VO_2_max during the squat task (4 to 9 min). BLa and %VO_2_max data averaged in stable condition, respectively. Furthermore, we examined significance of *r* on the relationships between %EMG_MVC_ of each muscle and MVC torque relative to body mass. The %EMG_MVC_ data averaged in stable condition in this analysis. Statistical significance was set at *p* < 0.05. All data were analyzed using statistical software (SPSS statistics 22; IBM, Tokyo, Japan).

## Results

The mean values for cardiorespiratory and metabolic parameters at LT during the incremental test were 2.3 ± 0.5 mmol/L for BLa, 46.5 ± 6.2% for %VO_2_max, and 65.8 ± 4.8% for %HRmax (Table 1).

### Cardiorespiratory response during body mass-based squat exercise

**Fig. 2:**
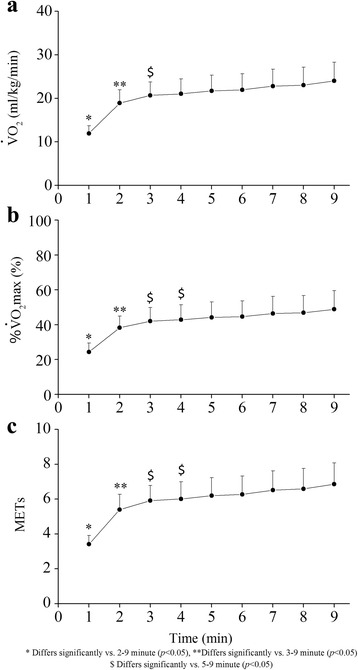
Oxygen uptake (VO_2_; **a**), percent maximal oxygen uptake (%VO_2_max; **b**) and metabolic equivalent (METs; **c**) during body mass-based squat exercise

VO_2_, %VO_2_max, and METs at 1 min from the exercise onset were significantly lower than those at other exercise time points, and significantly elevated until 4 min (*p* < 0.05). However, these variables did not significantly change after 5 min from the exercise onset (Fig. 2). The average values from 5 to 9 min were 22.7 ± 3.9 ml/kg/min for VO_2_, 46.2 ± 9.7% for %VO_2_max, and 6.5 ± 1.1 for METs. Time courses of VCO_2_ and VE were similar to those of VO_2_ and %VO_2_max. The average values across 5–9 min were 1.6 ± 0.4 L/min for VCO_2_ and 35.0 ± 8.8 L/min for VE. RQ significantly increased until the second time point (*p* < 0.05), but did not change from 3 to 9 min (1.1 ± 0.1).

**Fig. 3:**
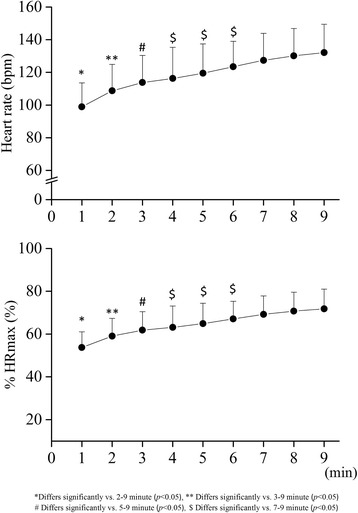
Heart rate (HR) and HR relative to HRmax (%HRmax) during body mass-based squat exercise

Time courses of HR and %HRmax during body mass-based squat exercise are presented in Fig. 3. HR and %HRmax at 1 min from the exercise onset were significantly lower than those at other exercise time points, and significantly elevated until 6 min (*p* < 0.05). In these variables, however, there were no significant differences in any combination across exercise time points during a duration from 7 to 9 min. The average values from 7 to 9 min were 131.1 ± 17.1 bpm and 71.2 ± 9.0%HRmax, respectively. %VO_2_max during the squat exercise across 5-9 min time points was negatively related to VO_2_max (*r* = −0.561, *p* < 0.05). The relationship between %HRmax during the squat exercise across 7-9 min time points and VO_2_max was not significant.

### BLa during body mass-based squat exercise

**Fig. 4:**
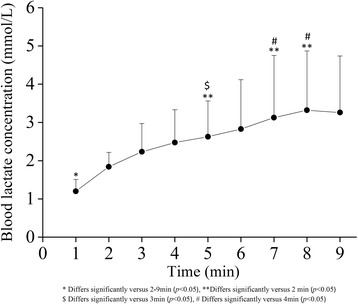
Blood lactate concentration (BLa) during body mass-based squat exercise

BLa was the lowest at 1 min during the exercise (Fig. 4, *p* < 0.05). BLa values at 2 and 3 min were lower than those at 5, 8, and 9 min (*p* < 0.05), respectively. There was no significant increase in BLa after 3 min from the exercise onset. The average value across 3–9 min was 3.6 ± 2.2 mmol/L. BLa during the body mass-based squat exercise across 3-9 min time points was negatively related to VO_2_max (*r* = −0.582, *p* < 0.05).

### %EMG_MVC_ during body mass-based squat exercise

**Fig. 5:**
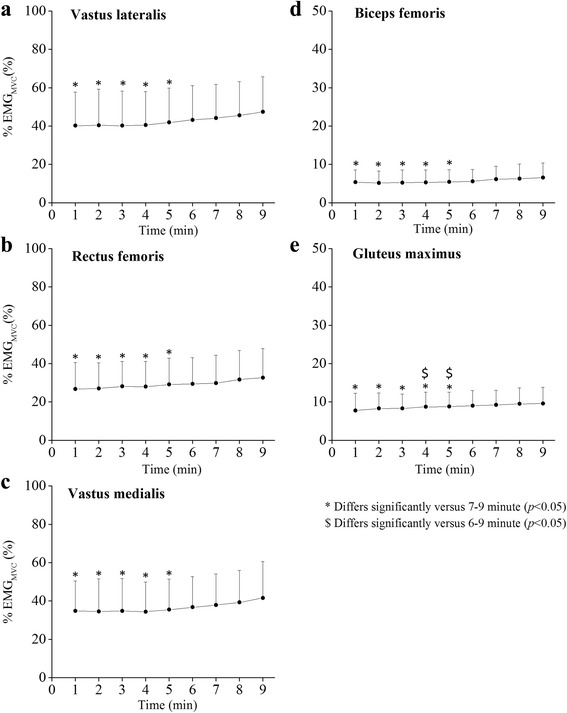
%EMG_MVC_ in lower extremity muscles during body mass-based squat exercise. **a** Vastus lateralis. **b** Rectus femoris. **c** Vastus medialis. **d** Biceps femoris. **e** Gluteus maximus

Time courses of %EMG_MVC_ during body mass-based squat exercise are presented in Fig. 5. The %EMG_MVC_ of VL, RF, VM, BF, and GM at 1 min from the exercise onset were significantly lower as compared to those at other exercise time points. These variables had no significant differences in any combination across exercise time points during a duration between 6 and 9 min. The %EMG_MVC_ of VL (45.0 ± 17.9%EMG_MVC_) and BF (6.3 ± 3.7%EMG_MVC_) is significantly higher after 6 min from exercise onset compared to those values across 1-5 min (40.6 ± 18.0%EMG_MVC_ for VL and 5.3 ± 3.2%EMG_MVC_ for BF). The %EMG_MVC_ values for RF, VM, and GM (31.4 ± 15.0%EMG_MVC_ for RF, 39.6 ± 17.3%EMG_MVC_ for VM, 9.3 ± 4.1%EMG_MVC_ for GM) were higher than those at 1-5 min (28.7 ± 13.4%EMG_MVC_ for RF, 34.8 ± 16.2%EMG_MVC_ for VM, 8.4 ± 4.0% for GM), respectively. The %EMG_MVC_ of each muscle was not significantly related to the cardiorespiratory parameters during the squat exercise. The %EMG_MVC_ for VL during the body mass-based squat exercise was negatively related to knee extension torque relative to body mass (KET/BM) (*r* = −0.627, *p* < 0.05). For RF and VM, the corresponding relationships were not significant (*r* = −0.175-0.268, n.s.).

## Discussion

The main findings of this study were that (1) cardiorespiratory and BLa significantly increased until 4 min from the start of body mass-based exercise, but did not further change after 5 min and (2) %VO_2_max and BLa during the squat exercise were negatively correlated with VO_2_max. These findings indicate that body mass-based squat exercise is conducted predominantly using an aerobic energy supply after 5 min from the start of the exercise, and that the magnitude of aerobic and anaerobic metabolisms during the exercise depends on an individual’s maximal aerobic power.

VO_2_max, VE, and BLa during the squat exercise did not significantly change after 5 min from exercise onset. Respiratory response is related to increases in metabolic products such as carbon dioxide and blood lactate in active muscle [27, 28] These metabolic products stimulate peripheral and central chemoreceptors and occurs ventilatory facilitation [27]. Considering earlier findings, the lack of significant changes in VO_2_max and VE after 5 min from exercise onset may be due to the fact that BLa did not change over the corresponding periods. Furthermore, low or moderate effort exercise can be maintained for a relatively long time, because blood lactate concentration is an equilibrium between rate of lactate appearance into and disappearance from the blood during exercises [9, 10], indicating that energy for the exercise is supplied largely via aerobic metabolism [29].

Oxygen uptake averaged across 5-9 min from exercise onset was 22.7 ± 3.9 ml/kg/min (46.2%VO_2_max). This value is equivalent to that for squat exercise at LT intensity (25 ml/kg/min) [8]. Furthermore, %VO_2_max at LT during the incremental loaded bicycle test was 46.5 ± 6.2%, being similar to that obtained during the body mass-based squat exercise. The VO_2_ during the body mass-based squat exercise (22.7 ± 3.9 ml/kg/min) was similar to another value obtained during half squat exercise with a load of approximately 30%1RM (18.3 ml/kg/min) [5]. This indicates that cardiorespiratory response to the body mass-based squat exercise is similar to that for squat exercise with an LT intensity external load. METs during the body-mass based squat exercise was 6.5 ± 1.1, corresponding to moderate aerobic exercise such as jogging and moderate aerobic dancing [30]. Taken together, we may say that, from the viewpoint of cardiorespiratory response, body mass-based squat exercise becomes a moderate intensity task when it is continued for at least for 5 min.

%VO_2_max and BLa during the body mass-based squat exercise were both significantly related to VO_2_max. This result indicates that oxygen uptake and metabolic response during the squat exercise may depend on maximal aerobic power. In other words, these current results imply that a person with higher VO_2_max can conduct body mass-based squat exercise with relative low physiological load. Higher aerobic power may be attributed to greater mitochondrial numbers, greater mitochondrial density, and higher oxidation ability in active muscle [31]. In the present results, however, the VO_2_ during the squat exercise was not significantly related to VO_2_max (*r* = 0.010,*p* = 0.970). This implies that the absolute oxygen uptake during the exercise is almost the same regardless of the magnitude of VO_2_max.

The mean value of %EMG_MVC_ during the squat exercise was 40.6% for VL, 28.7% for RF, 34.8% for VM, 5.3% for BF, and 8.4% for GM. These values are equivalent to those reported by Isear et al. [15] who examined EMG activities during body mass-based squat exercise (33.1%EMG_MVC_ for VL, 40.0%EMG_MVC_ for RF, and 10.8%EMG_MVC_ for GM). In the present study, the %EMG_MVC_ during the squat exercise significantly increased after 6 min from exercise onset. Because oxygen uptake is positively related to integrated EMG activity during an incremental loaded bicycle test [32], increases in integrated EMG, being derived from the number of action potentials and contracting muscle fibers [33], may be associated with elevated oxygen uptake during exercises. However, in the present results, the %EMG_MVC_ during the squat exercise increased in the later stages of the exercise, whereas oxygen uptake did not significantly shift after 5 min in this study. This might be due to the degree of increases in muscular activities during the exercise. In fact, the differences between the values averaged after 6 min and the values averaged over 1-5 min were 3.5-7.6%EMG_MVC_ for each muscle, indicating relatively small.

The %EMG_MVC_ for VL during body mass-based squat exercise was significantly related to KET/BM. This result supports our previous findings that the muscular activity levels of lower limb muscles during body mass-based exercises are negatively related to knee extension torque relative to body mass [11, 34]. This implies that relative effort during body mass-based squat exercise is greater in a person with low force-generating capacity than one with high force-generating capacity. Considering that increase in relative load during the incremental loaded bicycle test linearly elevates oxygen uptake and integrated EMG activity [32], %VO_2_max would be expected to depend on the muscular activity level during the squat exercise. However, the corresponding relationships were not significant in this study. Therefore, at least in young men, cardiorespiratory responses during body mass-based squat exercise may be independent of the muscular activity level of the knee extensors.

Because the design of this study was cross-sectional study, it is unclear whether body mass-based squat exercise training improves systemic and local endurance capacities. Miyamoto et al. [2] revealed that a 4-week intervention with low-intensity electrical muscle stimulation during knee extension exercises, corresponding to 3-4 METs, enhanced oxygen uptake during cycle ergometer. In the present study, METs during the body mass-based squat exercise reached 6.5 ± 1.1, and mainly activated the knee extensors. Body mass-based squat exercise may be one feasible approach for improving systemic and local endurance capacity when it is performed continuously at least for 5 min.

## Conclusions

From the viewpoint of cardiorespiratory and metabolic responses, body mass-based squat exercise is moderate intensity and is performed via aerobic metabolism after 5 min from exercise onset. Furthermore, the relative intensity of the squat exercise partially depends on the individual’s aerobic maximal power.

## Abbreviations

%EMG_MVC_: EMG amplitude relative to EMG_MVC_
%HRmax: HR relative to HRmax
%VO_2_max: VO_2_ relative to VO_2_max
ANOVA: Analysis of variance
BF: Biceps femoris
BLa: Blood lactate concentration
EMG: Electromyogram
EMG_MVC_: EMG amplitude during MVC
GM: Gluteus maximus
HR: Heart rate
HRmax: Maximal heart rate
KET: Knee extension torque
LT: Lactate threshold
METs: Metabolic equivalents
MVC: Maximal isometric voluntary contraction
RF: Rectus femoris
RMS: Root-mean-square
RPE: Rating of perceived exhaustion
RQ: Respiratory exchange ratio
VCO_2_: Carbon dioxide production
VE: Minute ventilation
VL: Vastus lateralis
VM: Vastus medialis
VO_2_: Oxygen uptake
VO_2_max: Maximal oxygen uptake
